# Exploratory Behaviours of Primitive Horses Based on Konik: A Preliminary Study

**DOI:** 10.3390/ani11030796

**Published:** 2021-03-12

**Authors:** Ewa Jastrzębska, Joanna Sadowska, Elżbieta Wnuk-Pawlak, Monika Różańska-Boczula, Iwona Janczarek

**Affiliations:** 1 Department of Horse Breeding and Riding, University of Warmia and Mazury in Olsztyn, Oczapowskiego 5, 10-719 Olsztyn, Poland; e.jastrzebska@uwm.edu.pl (E.J.); sad.joanna@wp.pl (J.S.); 2 Department of Horse Breeding and Use, University of Life Sciences in Lublin, Akademicka 13, 20-950 Lublin, Poland; iwona.janczarek@up.lublin.pl; 3 Department of Applied Mathematics and Computer Science, University of Life Sciences in Lublin, Akademicka 13, 20-950 Lublin, Poland; monika.boczula@up.lublin.pl

**Keywords:** Konik horses, exploration, behavioural tests

## Abstract

**Simple Summary:**

The behaviour of horses has evolved to ensure survival in emergency situations. The specific behaviour of horses is mainly determined by their instincts as an internal pressure to satisfy a specific need. Instinctive behaviours can include avoidance or flight as well as curiosity and the urge to explore. Exploratory behaviours can provide information about food, shelter, a new escape route, or a convenient place to raise offspring. However, this may come at the cost of a possible predator attack, isolation from the group, or injury in an accident in unknown territory. Standard behavioural tests can describe the need for exploration in horses and the severity of exploratory behaviour in a variety of situations. The experiment was carried out in two groups of Konik horses kept in a stable group and in a free-range group. The tested horses can be regarded as exhibiting the urge to explore, although their behavioural responses are individual and stimulus dependent.

**Abstract:**

This study aimed at assessing the behaviour of Konik geldings and mares, kept in a stable and in a free-range system, during behavioural tests regarded as a determinant of the exploration urge. A total of 19 Konik horses kept in individual stables and in a free-range system were included in the study. The experiment was conducted in five phases separated by five-day breaks. A one-stage passive human test was performed during the first phase, a three-stage active human test—2nd phase, a three-stage unknown object test—3rd phase, a two-stage unknown surface test—4th phase, and a one-stage test of social isolation—5th phase. Ten attributes were analysed, including the horse sex and the keeping system. The results were also correlated with one another. Konik horses were found to show the urge to explore, although their behavioural responses are individual and stimulus dependent. In many cases, the horse sex and the keeping system influence the exploratory behaviour, although it is manifested by a greater intensity in geldings than in mares, and in free-range horses than in those kept in a stable. The study is regarded as preliminary due to the small number of horses in the study groups.

## 1. Introduction

The horse’s species-specific behaviour developed to ensure their survival when under threat [1]. According to Peters et al. [2], the specific behaviour of a horse is mainly determined by its instincts as an internal pressure to satisfy a specific need. Aversive drives provoke stimulus avoidance, thereby protecting the animal from danger [3,4]. Appetitive drives, with their persistent striving to achieve a goal, are the opposite. Drives are behind instinctive behaviour [5]. Considerations of the stimulus-driven behavioural pattern of Equidae cannot leave out a strong urge to explore, which arises from these animals’ innate proneness to examine the surroundings [6]. The horse satisfies its urge to explore mainly with its sight, smell and hearing senses [7]. A strong urge to explore often encourages horses to actively examine new objects and situations [8].

According to Krueger et al. [9] and Hildebrandt et al. [10], the urge to explore diminishes with age, hence curiosity and exploratory behaviours are best developed in foals. Exploratory behaviour manifests as watching, smelling, licking and gnawing at unknown objects, as well as in exploratory movements, such as knocking, digging and pushing with a leg, and in wandering. Sometimes a new stimulus provokes such responses as the raised head, eyes wide open, looking ahead, ears pointed stiffly forward, dilated nostrils, tense muscles and the tail raised high [11]. The intensity of such responses usually depends on the type and distance from the object and a surprise effect [12]. When the situation is safe again, the horse resumes the interrupted activity. When uncertain, a horse may approach the unknown object, keeping a safe distance, and may make physical contact with it in an effort to explore it. Snorting may also appear as a consequence [13]. In general, it is assumed that exploratory activity is affected by the environment and it combines the multifactorial interactions present in it [14]. Unlike instinctive behaviour, exploratory behaviours are not cyclic in nature [15].

According to Safryghin et al. [16], exploratory behaviour includes elements of various forms of behaviour and it is not always possible to explain clearly what causes it. No publications describing the consequences of exploratory behaviour or the benefits that animals derive from exploration have been found. However, exploratory activity certainly results in the constant acquisition of information about the surroundings. Exploratory behaviour results, among others, in obtaining information about food, shelter, a new escape route or a convenient place for rearing offspring. However, this may come at the cost of a possible predator’s attack, isolation from the group or being hurt in an accident on unknown ground. Pisula [15] stresses that it is easy to identify the costs of exploratory behaviour but much more difficult to identify the gains. According to Suprun and Stanier [17], exploration of the surroundings brings more benefits to wild horses, as it not only helps them to gain experience but, owing to it, they can gain new resources and avoid spending energy on an unnecessary escape. According to Hildebrandt et al. [10], exploratory behaviour manifested as covering some distance does not depend on either the horse breed or sex. However, its intensity is affected by the knowledge of the surroundings. Exploration is affected in a positive and irreplaceable way by the possibility of maintaining relations with other horses in a herd, which was stressed by Baragli et al. [18].

The temperamental features of horses are of great importance in their use, which directly affects the safety of work with horses and their welfare. The use of several behavioural tests showed fearfulness and “reactivity-to-humans” in horses [19,20,21] that strongly impact their usability [22]. As a social prey animal, horses are particularly sensitive to separation from herd mates [23], as well as to novel and suddenly moving objects [19]. The violent reaction of the horse to isolation from conspecifics and to novel static or moving objects, which routinely occurs while riding horses, may jeopardize the safety of the riders. The tests developed to measure horses’ reactivity in these two situations (fear tests and separation tests) were used in the appraisal of equine temperamental traits [19,23,24,25,26].

Regarding the utility value of horses, it should be emphasised that a possibility of starting an exploratory activity is associated with a reward, so it can be used as reinforcement in the conditioning of instrumental responses [8]. Horses’ urge to explore correlates with behavioural and physiological responses to stimuli, which gives more information about the process of habituating or desensitizing horses to disturbing stimuli [27]. Due to various behavioural and physiological responses, the reactivity and emotional responses are different in horses taking part in different riding events. Therefore, the exploratory need level can determine the direction of horse training, which makes a study of it both cognitive and practical in horse use.

Konik horses were selected for the experiment because they are a breed that exhibits many of the behaviours typical of primitive horses. Studying their behaviour is additionally valuable as they have been introduced to various environments to maintain biodiversity and have been used in environmental projects in a few European countries. The breeding program aims to preserve these primitive, robust horses [28].

The hypothesis was adopted in the study that exploratory behaviour of Konik horses under an influence of stimuli depends on the horses’ sex and type of conditions they are kept in. It was assumed that both males and horses kept in stables should manifest a greater urge to explore than females and free-range horses. The authors of the present study assumed that male individuals are more interested in the exploration of the environment than mares which, in turn, are more focused on caring for their offspring. It was further adopted that horses kept in a stable system, where they are deprived of a range of natural stimuli, are more inclined to explorative behaviours to satisfy their curiosity. Horses’ urge to explore can be determined with various behavioural tests. They are based on a conflict of curiosity, triggering movements towards a stimulus, which is hindered by fear that motivates a horse to avoid the stimulus [17,29].

Therefore, this study aimed at assessing the behaviour of Konik geldings and mares, kept in a stable or in a free-range system, during standard behavioural tests regarded as a determinant of the urge to explore. The research used slightly modified behavioural tests used by Górecka-Bruzda et al. [30].

## 2. Materials and Methods

### 2.1. Animals

The studied animal group comprised 9 geldings and 10 mares of Konik, all horses at the age of 6–10 years. The experiment was attended by two women who were strangers to the experimental horses (to exclude the situation of learned obedience towards acquaintances). All the horses had been trained to work under the saddle, but they had not been used in this way for 12 months before the study started. They were clinically healthy during the experiment. The mares did not have their offspring near them, but they were all foaled (gestation 4–5 months). Seven of the horses (four geldings and three mares) had been kept only on pasture for at least 12 months (the free-range group). The other horses had been kept on pastures or in free-range during daylight and they were subsequently kept in 3 × 3 m stalls in a stable (the stable group). They had a manger in a corner, an automatic drinking bowl and a salt lick. The stall floor was bedded with wheat straw. During the pasture period (May–September), the horses kept in a stable were fed with hay only in the evenings (3 kg/horse). Hay was given to all horses in the mornings (2 kg/horse) and evenings (4 kg/horse) outside the pasture period. This keeping system had been applied for at least 12 months.

Unfortunately, it was not possible to include free-living horses from the reserve in the experiment. Due to their wild lifestyle, they are not used to any form of cooperation with humans, so it was not possible to conduct behavioural tests with their participation.

### 2.2. Experiment

The experiment was conducted at the Konik stud farm, located in the Lubelskie Voivodeship (Poland) on afternoons in late July and early August. Each of the five tests was performed on a different day. The subsequent days of testing were separated by five-day breaks. The free-range horses were isolated from other pastures on a 50 × 60 m pasture near the stables three days before the experiment started. The pasture was adjacent to a pasture of the same size where the stable horses were released. For the experiment purposes, a 10 × 15 m square was delineated, 10 m away from the short side fence of these pastures and the remaining area next to the pastures. The following weather conditions prevailed during the experiment: sunny or a little cloudy, air temperature: 22 °C ± 2.12; relative air humidity: 59% ± 3.13, atmospheric pressure: 998.36 hPa ± 5.22, wind velocity: 1.12 m/s ± 0.14.

One behavioural test was conducted on each experiment day (Table 1). For this purpose, the behavioural tests performed by Górecka-Bruzda et al. [30] were modified.

Test 1 (Passive human test): The person doing the test approached the horse’s left side and stopped 3.0 m from it, positioning themselves at an angle of 90 degrees relative to the longitudinal axis of the horse’s body. The person remained passive for five minutes, not trying to establish any contact with the horse. The time was measured (in seconds) counting from the test start, after which the horse came up to the person. Three hundred seconds was regarded as the maximum time. The time was automatically noted as the result for horses which failed the test. It was assumed that a higher test result (the longer time needed for a horse to come up to the person) reflected a lower urge to explore.

Test 2 (Active human test): The person conducting the test approached the horse’s left side and stopped 0.5 m from it, facing the longitudinal axis of its body. After 60 s they stroked it in the withers area (stage 2.1), then in the forehead area (stage 2.2) and finally in the muzzle area (stage 2.3). The stroking lasted 60 s in each stage. Whenever the horse did not allow the person to stroke a part of its body (e.g., backing off, putting back ears, threatening with teeth) the person attempted to stroke the next part. When the horse went away during the test stage, the person came up to it, stopped 0.5 m from it and proceeded to the next stage. The horse’s behaviour was observed during each stage of the experiment. Three separate grades were given on the basis of these observations, on the four-point scale designed by the author:

- 4 points: the horse does not walk away from the person, allows the person to touch it, does not exhibit undesirable behaviour, such as: putting back ears, wagging or tucking the tail, avoiding touch, biting, kicking, running away, hitting the ground with a leg;
- 3 points: the horse does not walk away from the person, initially it does not allow the person to touch it, puts back its ears, avoids being touched, hits the ground with a leg, but does not try to bite or kick;
- 2 points: the horse does not walk away from the person, but it does not allow the person to touch it, puts back its ears, avoids being touched, hits the ground with a leg, tries to bite or kick;
- 1 point: the horse walks away from the person when the latter tries to touch it.

It was assumed that the higher the score in each stage of the test, the greater the urge to explore.

Test 3 (Unknown object test): A horse in a stable headcollar was walked on a leading rope from the starting point at the long side of the arena (“Point A”) towards an orange rubber ball, 100 cm in diameter (“Unknown object”), which was at the distance of 15 m from “Point A”. The horse was led in such a way that it was walking away from the herd on the pasture when approaching the “Unknown object”. The person leading the horse gave a general direction, not making the animal walk at any specific speed and allowed the horse to stop. Subsequently, without using any force, the person using her voice or body language encouraged the horse to come as close as possible to the “Unknown object”. The maximum time of making a horse approach the “Unknown object” was 300 s.

Stage 3.1: The distance from the “Unknown object” was determined at which the horse first stopped while being led. A measuring tape was used (with an accuracy of 1.0 cm) to measure the distance between the front edge of the hoof which was the nearest to the “Unknown object” and the object. The first-stop distance was graded on a 5-point scale:

- 5 points: distance 0.00–0.50 m;
- 4 points: distance 0.51–1.00 m;
- 3 points: distance 1.01–1.50 m;
- 2 points: distance 1.51–2.00 m;
- 1 point: distance over 2.00 m.

It was assumed that the lower the score in this stage of the test, the smaller the urge to explore.

Stage 3.2: The distance (m) at which the horse ultimately stopped when approaching the “Unknown object”.

It was assumed that the higher the score in this stage of the test, the smaller the urge to explore.

Stage 3.3: It was assessed whether the “Unknown object” was examined by the horse by touch:

- 2 points: clear urge to explore: ears pointed towards the “Unknown object”, movements smooth and calm, examining the object with a leg or muzzle, muscles relaxed, tail falling freely or raised slightly;
- 1 point: no clear urge to explore: the horse was moving slowly, ears slightly pointed backwards, muzzle shut tight, distinct muscle tension, multiple stops, attempts at withdrawing or running away.

It was assumed that the lower the score in this stage of the test, the smaller the urge to explore.

Test 4 (Unknown surface test): An attempt was made to lead a horse over a blue tarpaulin fixed to the ground, dimensions: 3 × 5 m (“Unknown surface”). Due to the matt dimensions, it could not be walked around or jumped over. The front matt edge was placed 5 m from the starting point of the long side of the arena. A horse in a headcollar was walked on a leading rope in such a way that it was walking away from the herd on the pasture when approaching the “Unknown surface”. The person leading the horse gave a general direction, not making the animal walk at any specific speed and allowed the horse to stop. Subsequently, without using any force, and without any aids, the person encouraged the horse to come as close as possible to the “Unknown surface”. The maximum time of encouraging a horse to approach the “Unknown surface” was 300 s.

Stage 4.1: A 5-point scale was developed in order to classify the distance to the “Unknown surface”, at which the horse first stopped. A measuring tape was used (with an accuracy of 1.0 cm) to measure the distance between the front edge of the hoof which was the nearest to the “Unknown surface” and the central point of the surface:

- 5 points: distance 0–0.50 m;
- 4 points: distance 0.51–1.00 m;
- 3 points: distance 1.01–1.50 m;
- 2 points: distance 1.51–2.00 m;
- 1 point: distance of 2.00 m.

It was assumed that the lower the test score, the smaller the urge to explore.

Stage 4.2: the time (s) was measured after which the horse decided to cross the “Unknown surface”. If the horse did not cross it within 300 s, the test result was regarded as negative, i.e., the maximum time of 300 s was recorded. It was assumed that the higher the test result, the smaller the urge to explore.

Test 5 (Social isolation test): A horse was walked along a dirt road to a distance of 60 m away from the pasture gate, where the rest of the herd was staying, and then stopped for 60 s at a place where it could not have eye contact with the other horses. In the next step, the horse was unfastened from the leading rope and allowed to return to the herd. When being walked away from the herd, the horse’s behaviour was assessed on a 4-point scale:

- 4 points: The horse moves at an even pace over a whole distance, it does not stop or manifest any behaviour typical of social isolation: vocalisation, intensified body movements, tense muscles, raised head, eyes wide open, head turned back to look at the herd, ears pointed stiffly forward, muzzle dilated and tail raised high;
- 3 points: The horse moves slowing down and accelerating for at least 50% of the distance, it does not stop, exhibits (at least one type of) behaviour typical of social isolation: vocalisation, intensified body movements, tense muscles, raised head, eyes wide open, head turned back to look at the herd, ears pointed stiffly forward, muzzle dilated, tail raised high;
- 2 points: The horse moves, slowing down and accelerating, for at least 25% of the distance, it stops at least once, exhibits (at least two types of) behaviour typical of social isolation: vocalisation, intensified body movements, tense muscles, raised head, eyes wide open, head turned back to look at the herd, ears pointed stiffly forward, muzzle dilated, tail raised high;
- 1 point: The horse does not move or moves slowing down and accelerating for less than 25% of the distance, exhibits (at least three types of) behaviour typical of social isolation: vocalisation, intensified body movements, tense muscles, raised head, eyes wide open, head turned back to look at the herd, ears pointed stiffly forward, muzzle dilated, tail raised high.

It was assumed that the lower the test score, the smaller the urge to explore. All behavioural activities and their definitions are presented in Table 2.

### 2.3. Statistical Analysis

The aim of the study was to examine to what extent the independent variables (sex, method of keeping) affect the responses in behavioural tests (Test 1–Test 5) performed on Konik horses. The experimental data were analysed with the non-parametric U Mann–Whitney test due to deviations from normality (Shapiro–Wilk test) and homoscedasticity (Levene test). Statistically significant results (*p* < 0.05) were also presented in graphs, which showed the diversification of the groups under study. Links between the parameters under study (tests) were also examined with the Spearman rank correlation. All of the calculations and graphs were generated with the Statistica 13.1 software package (StatSoft2013, www.statsoft.com/textbook/, accessed on 18 February 2018).

A preliminary analysis of the experimental data with the Shapiro–Wilk test verified the assumption of the variable distribution normality within the groups under study, whereas the Levene test verified the equality of the variable variance between the groups. The null hypothesis was not rejected (*p* > 0.05), i.e., the distribution normality and homoscedasticity were confirmed, only for observations regarding the time after which the horse decided to cross the unknown surface. Therefore, the non-parametric U Mann–Whitney test was performed in order to examine the impact of sex and method of horse keeping on the behavioural test results.

## 3. Results

In order to provide general characteristics of the horses’ behaviour during the tests, the mean results of the behavioural tests were presented together with the Min/Max values, standard deviation and with the mean expressed as a percentage of the maximum value (Table 3).

The probabilities (*p*-Value) are presented in Table 4. The analysis showed that the sex significantly differentiated the time after which the horse approached the passive human (Test 1) and the distance from the central point of the unknown surface at which the horse stopped (Test 4, stage 4.1). The keeping method had a significant impact on the results in the third stage of the active human test, i.e., during the muzzle stroking (Test 2, stage 2.3), and in the social isolation test (Test 5).

**Figure 1:**
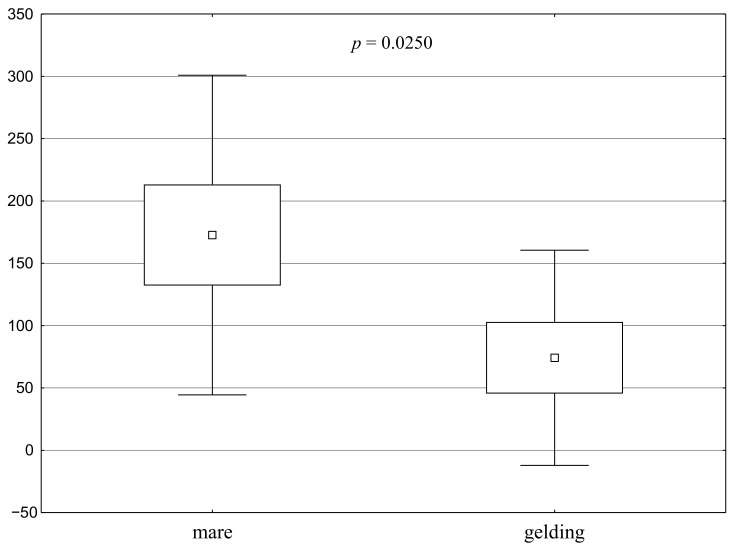
Distribution of Test 1 results for the groups—Passive human test: time after which the horse comes up to the person (s) (a square shows the mean, a box corresponds to the range of the mean ± standard error, and “whiskers” show the range of the mean ± standard deviation). A significant difference between the groups is confirmed by the value of *p* < 0.05.

**Figure 2:**
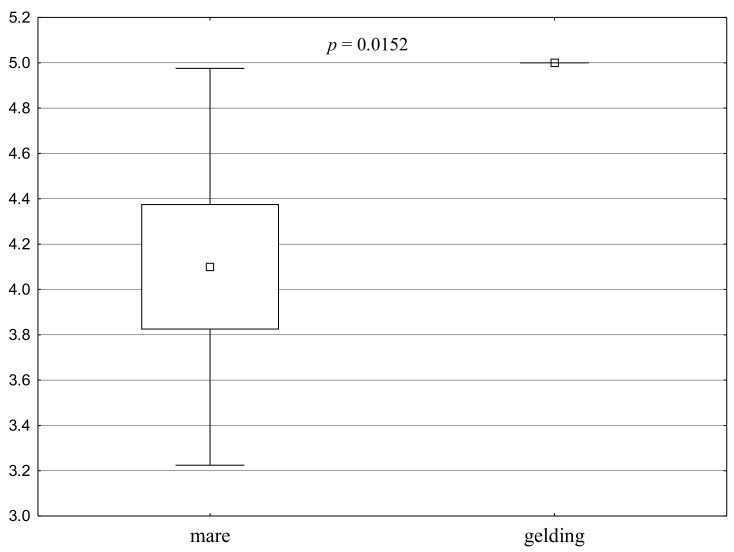
Distribution of Test 4 results for the groups—Unknown surface test, stage 4.1: points for the distance from the new surface at which the horse first stopped (a square shows the mean, a box corresponds to the range of the mean ± standard error, and “whiskers” show the range of the mean ± standard deviation). A significant difference between the groups is confirmed by the value of *p* < 0.05.

**Figure 3:**
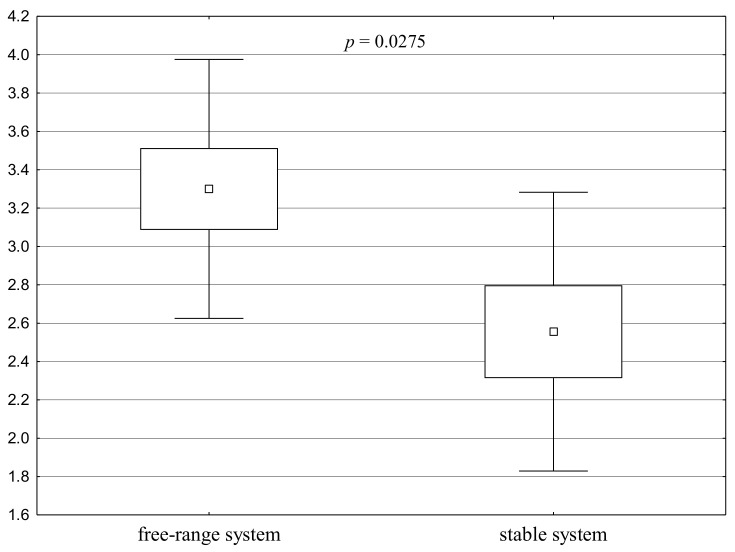
Distribution of Test 2 results for the groups - Active human test, stage 2.3: points for stroking the muzzle (a square shows the mean, a box corresponds to the range of the mean ± standard error, and “whiskers” show the range of the mean ± standard deviation). A significant difference between the groups is confirmed by the value of *p* < 0.05.

**Figure 4:**
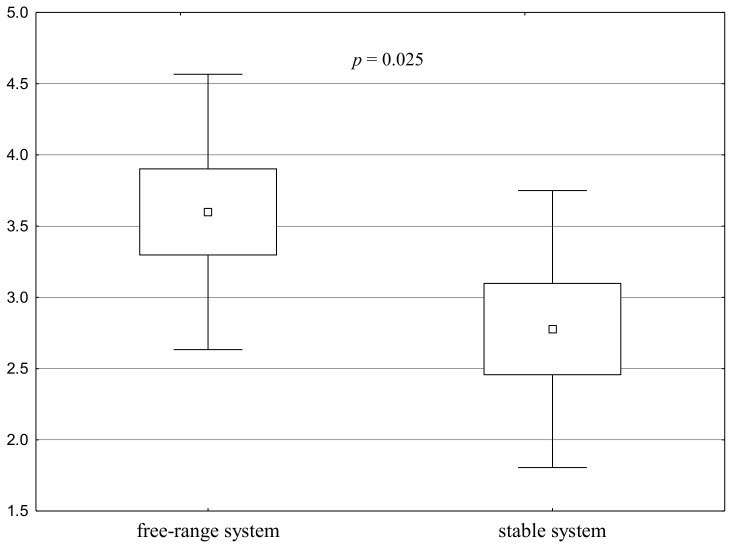
Distribution of Test 5 results for the groups—Social isolation test: points for the will to walk away from the other horses (a square shows the mean, a box corresponds to the range of the mean ± standard error, and “whiskers” show the range of the mean ± standard deviation). A significant difference between the groups is confirmed by the value of *p* < 0.05.

The statistically significant results (*p* < 0.05) were also presented in the charts and show the direction of differentiation of the studied groups (Figure 1, Figure 2, Figure 3 and Figure 4). The time after the mares came up to the person in the passive human test (Test 1) was significantly longer than that time for geldings, both for stable and free-range mares (Figure 2). There was a 45% difference between geldings and mares in this regard in the group of horses kept in a stable. The difference between geldings and mares in the free-range group was 57%.

The mares received significantly lower grades than geldings in the unknown surface test, in stage 4.1, in which points were given for the distance from its central point at which the horse first stopped (Figure 2). All the geldings received the highest grade regardless of the keeping system. The grades in the mare group were 24% lower for mares kept in a stable and 10% lower for mares in the free-range group.

The grades in the free-range group were significantly higher than those in the stable group in the active human test during the muzzle stroking stage (Test 2, stage 2.3) for both geldings and mares (Figure 3). The differences were particularly visible for geldings. The score for geldings in the free-range group was 63% higher than the score for geldings in the stable group.

Spearman rank correlation coefficients were determined to examine the association between the attributes in Tests 1–5. The results are presented in Table 5. Two significant positive correlations and two significant negative correlations were found. The positive correlations were recorded between the time after which the horse came up to the person and the distance at which it ultimately stopped when approaching the unknown object, and between the score for stroking the forehead and score for stroking the muzzle. Negative correlations were found between the time after which the horse came up to the person and score for touching the unknown object with the muzzle, and between the distance at which it ultimately stopped when approaching the unknown object and the score for touching the unknown object with the muzzle.

## 4. Discussion

The results of the tests performed on Konik horses were diverse and, for some tests, indicated a significant urge to explore. The results of tests based on an animal’s response to a human showed that the horses needed about half of the maximum time to come up to an immobile person. In the conducted experiment, the scores for stroking specific parts of the horse’s body were always higher than 70% of the maximum number, which means that most horses did not mind stroking and did not exhibit any responses recognised by horse users as undesirable, such as putting back ears, wagging or tucking the tail, biting, running away, stirring, hitting the ground with a leg or just avoiding the touch. Therefore, it can be suggested that curiosity outweighs fear, but the balance between these features is an individual feature, which is indicated by the standard deviation of the studied attributes. The results are probably due to the fact that all the horses had had contact with people when saddled and being groomed. Similar suggestions were presented by Hall et al. [31], who pointed out that horse training had a long-term impact on their behaviour in horse–human relations. Therefore, it could be assumed that the test results for horses living in the wild without human interference would be completely different [32,33].

New object tests provided results indicating the urge to explore. However, it cannot be excluded that the horses were coming closer to unknown objects because they trusted or obeyed the person who was leading them. However, it was a substantial problem for them to cross the unknown surface. In these cases, fear usually outweighed curiosity, which indicates that the response to a stimulus depends mainly on its type. A similar view was expressed by Pierard et al. [12], who characterised diverse responses to stimuli in horses used by the mounted police.

In a detailed analysis of the results, the sex factor impact was observed only in two out of the ten attributes under analysis and the horse-keeping system factor—in another two. Most probably, it is the primitive nature of Konik horses that levels out these differences [28]. The results may have been affected by the fact that the unpredicted responses and relations in a horse herd are still regarded as not fully explained [34].

An analysis of the impact of sex factor showed that mares not only needed more time to establish interactions with an immobile person, but they did not come to the unknown surface as close as geldings did. These results may be a consequence of the functions played by mares in a horse herd [35]. They have to remain cautious to ensure safety to their present or future offspring and only then are eager to explore and examine unknown situations, which affects the time of response to danger. Watts et al. [36], as well as Budzynska and Krupa [37], stress that mares are generally cautious; they appear more comfortable in a herd accompanied by other horses, but they exhibit distinct caring behaviour instead. Pregnant mares are a specific group in this case, as they avoid risky situations instinctively, keeping a greater distance. Fenner et al. [38] stress that geldings are regarded as calm, reliable and eager to cooperate with people, without such intensive male behaviour as in the case of stallions.

Interestingly, the sex factor was not found to have any impact at any stage of the active human test, which is regarded as invading the personal space of a horse much more than the passive human test [20]. In consequence, it seems that the mares are more cautious when the person is immobile. Such immobility may be associated with a predator lurking before the attack. Similar observations were made by Proops et al. [39], who analysed horses’ responses to a human’s “body language”, which resembled an attack.

The method of horse keeping had an impact on the horses’ behaviour, which was confirmed by the results of the social isolation test (Test 5) and during the muzzle stroking in the active human test (Test 2, stage 2.3). Isolation had a smaller impact on free-range horses than on those kept in a stable. This finding is intriguing given the acute herd instinct stressed by Górecka-Bruzda et al. [28], which is apparent mainly in herds similar to natural. Therefore, it can be predicted that such instinct can be manifested in free-range horses, which live in herds with slightly upset social relations. The findings of the present study differ from those cited. These differences in the obtained results may be caused by emotional–behavioural disorders in horses kept in a stable, as indicated by studies conducted by Lesimple et al. [40]. The method of keeping horses in a stable may develop uncertainty in horses, which inhibits the urge for exploration. It seems that the answer to these questions cannot be given at this stage as there were too few horses under study, which the authors regard as a flaw of this research. However, the above considerations have been corroborated because, according to Hoffman et al. [41], keeping horses in a stable induces behavioural problems indicative of stress. Similarly, Löckener et al. [42] and Sigurjónsdóttir and Haraldsson [43] stress that the horse-keeping system affects horses’ behaviour. Horses kept in a stable system are more dependent on human care and less likely to make decisions than horses living in more natural conditions. Moreover, horses kept in a stable sometimes become mentally withdrawn, which is similar to depression diagnosed in people, leading to weaker responses to certain stimuli [44]. There are also animals described as being deprived of full herd relations, which makes them susceptible to general stress and even unable to defend themselves against predators [45]. In the study, the conditions of the isolation test were probably not ferocious enough to provoke behaviour associated with the herd instinct in the free-range horses. The stimulus may have been too weak.

Notably, free-range horses were not only less reluctant to walk away from the herd but were also more willing to allow their muzzle to be stroked than the horses kept in the stable. It was especially noticeable in the case of geldings. This may further indicate that geldings and free-range horses demonstrate a greater urge to explore than mares and horses kept in stables, which is not fully in line with the study hypothesis.

However, an analysis of the correlations between the test results does not reveal the extent of the urge to explore in Konik horses. This is because there are only 9% significant correlations between the attributes. The correlations showed positive associations between the passive human test and the new object test and between the score for muzzle stroking and forehead stroking in the active human test. Moreover, negative relationships were found between the passive human test and touching new objects with the muzzle and between three-stage results in the unknown object test. This indicates that Konik horses, which more willingly interacted with the passive human and touched the new object with the muzzle, tended to approach the new object and study it with interest from a closer distance. Admittedly, the coefficients, together with the interpretation of the increasing or decreasing test results clearly show the urge to explore or its absence in the Konik horses. However, the small number of such associations does not allow for generalising the results. In general, it can be claimed that the response of Konik horses to a considerable degree depends on the stimulus applied. Possibly with a larger group of experimental horses, more regularities could be observed. Mengoli et al. [14] examined the emotional balance in horses in learning and recalling tests, and their findings were similar.

## 5. Conclusions

Konik horses can be regarded as exhibiting the urge to explore, although their behavioural responses are individual and may vary depending on the applied stimuli. Presumably, when conducting a similar study on a larger group of horses it would be possible to observe more regularities and relationships. The horses’ sex and the keeping system have a minor effect on exploratory behaviour, although it is exhibited with a greater intensity in geldings than in mares, and in free-range horses than in those kept in a stable.

## Figures and Tables

**Table 1:** A list of behavioural tests performed.

Test Number	Test Name	Number of Test Stages	Symbols of the Assessment Stage in a Test
1	Passive human test	1	1
2	Active human test	3	2.1, 2.2, 2.3
3	Unknown object test	3	3.1, 3.2, 3.3
4	Unknown surface test	2	4.1, 4.2
5	Social isolation test	1	5

**Table 2:** Activities measured in social isolation test.

Activity	Description
Vocalisation	Neighing (at least once during the test), snorting (more than twice during the test)
Intensified Body Movements	Sudden head movements (more than twice during the test), kicking or hitting the ground with a leg (at least once during the test), rearing (more than twice during the test)
Tense Muscles	Visible trembling of shoulder region muscles unrelated to defending the body against insects (for at least 10 s during the test)
Raised Head	Mouth raising at least to the level of withers (for at least 10 s during the test)
Eyes Wide Open	Tense eyelids, visible white parts of the eye (for at least 10 s during the test)
Head Turned Back to Look at the Herd	Looking back, looking for the herd, the neck bent at an angle of at least 45 degrees relative to the normal position (more than twice during the test)
Ears Pointed Stiffly Forward	Ears pricked up pointing forwards and stationary for 3 s or more (more than twice during the test)
Muzzle Dilated	Dilated and tense muzzle for at least 5 s (more than twice during the test)
Raised Tail High	Fleshy part of tail outstretched horizontally or elevated above horizontal line (for at least 10 s during the test)

**Table 3:** Mean, minimum and maximum values and standard deviation (SD) for the subsequent tests and their stages.

*n* = 19	Mean	Min	Max	SD	Mean as % of Max Value
Test 1	126.05	10.00	300.00	118.69	42
Test 2, Stage 2.1	3.74	2.00	4.00	0.65	94
Test 2, Stage 2.2	3.32	2.00	4.00	0.82	83
Test 2, Stage 2.3	2.95	2.00	4.00	0.78	74
Test 3, Stage 3.1	4.32	1.00	5.00	1.11	86
Test 3, Stage 3.2	1.74	1.00	10.00	2.16	12
Test 3, Stage 3.3	1.84	1.00	2.00	0.37	92
Test 4, Stage 4.1	4.53	3.00	5.00	0.77	91
Test 4, Stage 4.2	219.79	15.00	300.00	80.08	73
Test 5	3.21	1.00	4.00	1.03	80

Test 1: Passive human test: time after which the horse comes up to the person (s). Test 2: Active human test, stage 2.1: points for stroking in the withers area (pts), stage 2.2: points for stroking the forehead (pts), stage 2.3: points for stroking the muzzle (pts). Test 3: Unknown object test, stage 3.1: points for the distance from the object at which the horse first stopped (pts), stage 3.2: the distance at which the horse ultimately stopped when approaching the unknown object (m), stage 3.3: touching the unknown object with muzzle (pts). Test 4: Unknown surface test, stage 4.1: points for the distance from the new surface at which the horse first stopped (m), stage 4.2: time after which the horse decided to cross the unknown surface (s). Test 5: Social isolation test: points for the will to walk away from the other horses (pts).

**Table 4:** Probability values (*p*-Value) adjusted for a one-way alternative hypothesis and a small group size (<20) the values statistically significant at 0.05 are in bold.

Source of Variation	Test 1	Test 2 Stage 2.1	Test 2 Stage 2.2	Test 2 Stage 2.3	Test 3 Stage 3.1	Test 3 Stage 3.2	Test 3 Stage 3.3	Test 4 Stage 4.1	Test 4 Stage 4.2	Test 5
Sex	0.0250	0.3121	0.4675	0.0957	0.3121	0.1442	0.1442	0.0152	0.5000	0.1265
Method of Keeping	0.3121	0.4032	0.2071	0.0275	0.1846	0.3121	0.3415	0.1352	0.2312	**0.0250**

Test 1: Passive human test: time after which the horse comes up to the person (s). Test 2: Active human test, stage 2.1: points for stroking the withers area (pts). Test 2: Active human test, stage 2.2: points for stroking the forehead (pts). Test 2: Active human test, stage 2.3: points for stroking the muzzle (pts). Test 3: Unknown object test, stage 3.1: points for the distance from the object at which the horse first stopped (pts). Test 3: Unknown object test, stage 3.2: the distance to which the horse approached the unknown object (m). Test 3: Unknown object test, stage 3.3: touching the unknown object with the muzzle (pts). Test 4: Unknown surface test, stage 4.1: points for the distance from the new surface at which the horse first stopped (m). Test 4: Unknown surface test, stage 4.2: time after which the horse decided to cross the unknown surface (s). Test 5: Social isolation test: points for the will to walk away from the other horses (pts).

**Table 5:** Spearman rank correlation coefficients (r_s_) for the results of subsequent tests and their stages (the values statistically significant at 0.05 are in bold).

(r_s_)	Test 1	Test 2 Stage 2.1	Test 2 Stage 2.2	Test 2 Stage 2.3	Test 3 Stage 3.1	Test 3 Stage 3.2	Test 3 Stage 3.3	Test 4 Stage 4.1	Test 4 Stage 4.2	Test 5
Test 1	-	−0.30	0.14	−0.16	−0.16	**0.59**	**−0.60**	−0.24	−0.10	−0.32
Test 2, Stage 2.1	−0.30	-	0.17	−0.03	0.37	−0.13	0.17	−0.06	0.17	−0.16
Test 2, Stage 2.2	0.14	0.17	-	**0.61**	0.09	−0.04	0.04	0.04	−0.16	−0.27
Test 2, Stage 2.3	−0.16	−0.03	**0.61**	-	−0.02	−0.37	0.35	0.08	−0.24	0.01
Test 3, Stage 3.1	−0.16	0.37	0.09	−0.02	-	−0.19	0.23	−0.14	0.34	0.16
Test 3, Stage 3.2	**0.59**	−0.13	−0.04	−0.37	−0.19	-	**−0.99**	−0.20	−0.08	−0.17
Test 3, Stage 3.3	**−0.60**	0.17	0.04	0.35	0.23	**−0.99**	-	0.22	0.08	0.14
Test 4, Stage 4.1	−0.24	−0.06	0.04	0.08	−0.14	−0.20	0.22	-	−0.17	0.13
Test 4, Stage 4.2	−0.10	0.17	−0.16	−0.24	0.34	−0.08	0.08	−0.17	-	0.10
Test 5	−0.32	−0.16	−0.27	0.01	0.16	−0.17	0.14	0.13	0.10	-

Test 1: Passive human test: time after which the horse comes up to the person (s). Test 2: Active human test, stage 2.1: points for stroking the withers area (pts). Test 2: Active human test, stage 2.2: points for stroking the forehead (pts). Test 2: Active human test, stage 2.3: points for stroking the muzzle (pts). Test 3: Unknown object test, stage 3.1: points for the distance from the object at which the horse first stopped (pts). Test 3: Unknown object test, stage 3.2: the distance to which the horse approached the unknown object (m). Test 3: Unknown object test, stage 3.3: touching the unknown object with the muzzle (pts). Test 4: Unknown surface test, stage 4.1: points for the distance from the new surface at which the horse first stopped (m). Test 4: Unknown surface test, stage 4.2: time after which the horse decided to cross the unknown surface (s). Test 5: Social isolation test: points for the will to walk away from the other horses (pts).

## Data Availability

None of the data were deposited in an official repository. The data are available upon request.
